# Micro-utrophin Improves Cardiac and Skeletal Muscle Function of Severely Affected D2/*mdx* Mice

**DOI:** 10.1016/j.omtm.2018.10.005

**Published:** 2018-10-16

**Authors:** Tahnee L. Kennedy, Simon Guiraud, Ben Edwards, Sarah Squire, Lee Moir, Arran Babbs, Guy Odom, Diane Golebiowski, Joel Schneider, Jeffrey S. Chamberlain, Kay E. Davies

**Affiliations:** 1 Oxford Neuromuscular Centre at the University of Oxford, Department of Physiology, Anatomy and Genetics, Oxford OX1 3PT, UK; 2 Wellstone Muscular Dystrophy Research Centre, Department of Neurology, University of Washington, Seattle, WA, USA; 3 Solid Biosciences, Cambridge, MA, USA

**Keywords:** Duchenne, utrophin, AAV, D2/*mdx*, cardiomyopathy, cardiac cine-MRI

## Abstract

Duchenne muscular dystrophy (DMD) is an X-linked muscle-wasting disease caused by mutations in the dystrophin gene. DMD boys are wheelchair-bound around 12 years and generally survive into their twenties. There is currently no effective treatment except palliative care, although personalized treatments such as exon skipping, stop codon read-through, and viral-based gene therapies are making progress. Patients present with skeletal muscle pathology, but most also show cardiomyopathy by the age of 10. A systemic therapeutic approach is needed that treats the heart and skeletal muscle defects in all patients. The dystrophin-related protein utrophin has been shown to compensate for the lack of dystrophin in the mildly affected BL10/*mdx* mouse. The purpose of this investigation was to demonstrate that AAV9-mediated micro-utrophin transgene delivery can not only functionally replace dystrophin in the heart, but also attenuate the skeletal muscle phenotype in severely affected D2/*mdx* mice. The data presented here show that utrophin can indeed alleviate the pathology in skeletal and cardiac muscle in D2/*mdx* mice. These results endorse the view that utrophin modulation has the potential to increase the quality life of all DMD patients whatever their mutation.

## Introduction

Duchenne muscular dystrophy (DMD) is a severe and progressive muscle-wasting disorder affecting 1:5,000 boys.^1^, ^2^, ^3^ DMD is caused by mutations in the dystrophin gene leading to the loss of dystrophin, a large structural protein located at the sarcolemma. In its absence, the sarcolemma is highly susceptible to contraction-induced injury causing muscle degeneration and replacement of contractile material with adipose and fibrotic tissue.^4^, ^5^ These processes typically lead to loss of ambulation between 8 and 12 years, and patients succumb to respiratory and/or cardiac failure in their second or third decade of life.^6^ DMD is primarily recognized as a skeletal muscle disorder, and historically, respiratory muscle insufficiencies accounted for the vast majority of patient deaths.^7^ However, with improved disease management, cardiomyopathy has emerged as a leading cause of death in patients receiving assisted ventilation.^8^ Despite recent progress and extensive research over the last three decades, there is still no effective treatment for DMD. Thus, development of treatments that target skeletal and cardiac muscle is essential for improving both quality of life and longevity of DMD patients.

We have focused on the development of utrophin modulation therapy using small molecules; this approach will potentially target the skeletal and cardiac muscle and is applicable to all patients irrespective of their mutation. Utrophin is a structural and functional autosomal paralog of dystrophin^9^, ^10^ that is localized to the sarcolemma during fetal development and confined to the neuromuscular junction (NMJ) and myotendinous junctions (MTJs) in mature muscle.^11^, ^12^ Utrophin is also expressed in regenerating myofibers in adult muscle.^13^, ^14^ Over the last two decades, we have shown in pre-clinical studies that both transgenic overexpression and pharmacological modulation of utrophin prevents skeletal muscle pathology in dystrophin-deficient (*mdx*) mice.^15^, ^16^, ^17^, ^18^, ^19^ Although we have shown the capacity of small compounds to induce utrophin expression in the hearts of *mdx* mice,^18^, ^19^ the functional impact of utrophin on the dystrophic myocardium still needs to be fully investigated.

Adeno-associated viral (AAV) vectors are well characterized as gene-therapy tools with the capacity for systemic delivery.^20^, ^21^ There are multiple serotypes of AAV that have different tissue tropisms; of these, AAV9 can target skeletal muscle and has shown the strongest transduction of the myocardium.^22^ As the 14-kb full-length dystrophin gene is too large to be packaged into an AAV vector (limited to ∼4.8 kb), truncated (“mini” and “micro”) dystrophin constructs have been developed and optimized for use in DMD.^23^, ^24^, ^25^, ^26^, ^27^, ^28^ Clinical trials investigating micro(μ)-dystrophin (Solid Biosciences; phase I and II; NCT03368742) and mini-dystrophin (Pfizer; phase I b; NTC03362502) for the use in DMD have been initiated.^29^, ^30^ Similar pre-clinical studies were also conducted using a μ-utrophin construct and were found to improve the pathology of dystrophin and utrophin double knockout (*dko*) mice^31^ and adenovirus-mediated utrophin gene transfer also mitigated the more severe pathological phenotype of golden retriever dogs with canine X-linked muscular dystrophy.^32^ Given the capacity of AAV to deliver a transgene to both cardiac and skeletal muscle, we employed an AAV9 serotype carrying the utrophin μ-gene to assess its impact on cardiac function and skeletal muscle pathology.

Most of the pre-clinical studies in the mouse have been carried out in C57BL/10ScSn-*Dmd*^*mdx*^/J (BL10/*mdx*) mice that show a relatively mild phenotype, particularly in the heart. In recent years, the D2.B10-*Dmd*^*mdx*^/J (D2/*mdx*) mouse model has been recognized as a potential pre-clinical model for DMD due to the lesser regenerative capacity of the D2/2J (D2/wild-type [WT]) parent strain.^33^, ^34^ Coley et al.^35^ and Vohra et al.^36^ showed that pathology in D2/*mdx* mice was more severe than age-matched BL10/*mdx* mice. Importantly, left ventricle (LV) systolic dysfunction has been shown in 6-month-old D2/*mdx* mice, whereas the equivalent cardiac defects are not present in BL10/*mdx* mice until 12 months of age.^37^ D2/*mdx* mice therefore offer a promising and more acutely affected model for cardiac studies. We therefore employed cardiac cine-MRI as it represents the most accurate, non-invasive approach for assessing cardiac function.^38^, ^39^ Of the parameters acquired by MRI, ejection fraction is the most accurate measure of systolic function and therefore was the primary parameter of interest.^39^

The aim of this work was to elucidate the functional impact of utrophin modulation on a more severe model of muscular dystrophy in mice. We explored whether μ-utrophin delivery could attenuate the pathology in skeletal muscle and tested the functional effects of utrophin expression on the dystrophin-deficient heart.

## Results

### 21-Week-Old D2/*mdx* Mice Exhibit Significant Cardiac Dysfunction Indicative of Hypertrophic Cardiomyopathy

**Figure 1:**
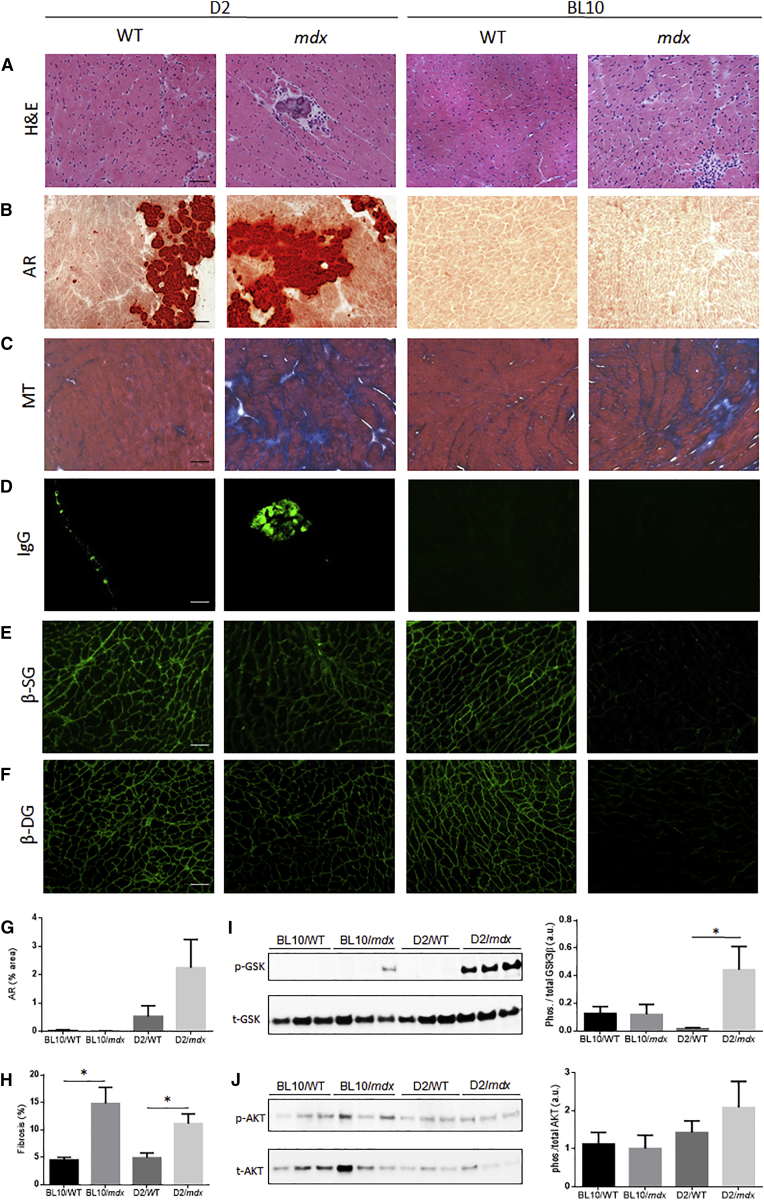
Characterization of Cardiac Cross-Sections from 21-Week-Old D2/WT, D2/*mdx*, BL10/WT, and BL10/*mdx* Mice Representative images of (A) muscle architecture (assessed by H&E), (B) calcification (red; assessed by alizarin red; AR), (C) fibrosis (assessed by Masson’s trichrome; MT), (D) IgG staining (green), (E) β-sarcoglycan (β-SG), and (F) β-dystroglycan (β-DG) immunofluorescence in cardiac cross-sections from 21-week-old mice. Quantification of (G) AR and (H) MT, expressed as percentage area. Representative images and quantification of protein expression of (I) phosphorylated and total GSK-3β (GSK) and (J) phosphorylated and total AKT assessed by western blot. Scale bars, 100 μm. Data represented as mean ± SEM, *p < 0.05 versus vehicle control mice, n = 6/group.

Cardiac cine-MRI assessment revealed significantly elevated LV stroke volume, right ventricle (RV) end diastolic volume, and RV stroke volume in 14-week-old BL10/*mdx* mice compared to age-matched BL10/WT mice (Table 1). No differences were observed between cardiac function of D2/*mdx* and D2/WT mice at 14 weeks of age (Table 1). Surprisingly, 21-week-old D2/*mdx* mice showed significantly elevated LV mass, LV ejection fraction, LV stroke volume and RV ejection fraction compared to D2/WT mice (Table 1). No differences were observed in cardiac function between BL10/WT and BL10/*mdx* mice at 21 -weeks of age (Table 1). At 28 weeks of age, no statistical differences were observed between D2/*mdx* and D2/WT mice in either LV or RV parameters (Table 1). A significant increase in heart mass normalized to body mass was observed in the hearts of 21-week-old D2/*mdx* mice compared to D2/WT mice (Table S1). These differences in heart mass were resolved by 28 weeks of age (Table S1). Skeletal muscle pathology of D2/*mdx* mice was consistent with previous reports^35^ (data not shown). As 21-week-old D2/*mdx* mice exhibited elevated systolic function compared to WT control mice, markers of pathology were assessed in this cohort. D2/*mdx* mice exhibited more prominent necrosis (Figure 1A), fibrosis (Figure 1B), calcification (Figure 1C) and immunoglobulin G (IgG)-positive regions (Figure 1D) as well as loss of components of the dystrophin glycoprotein complex (DGC) from the sarcolemma (Figures 1E and 1F) compared to age-matched WT controls. Quantification of alizarin red revealed a non-significant increase of calcification in D2/*mdx* mice compared to D2/WT mice (Figure 1G). Quantification of Masson’s trichrome revealed a significant increase of fibrosis in both BL10/*mdx* and D2/*mdx* mice relative to their respective WT control strains (Figure 1H). Phosphorylated glycogen synthase kinase (GSK)-3β and serine/threonine kinase (AKT) are associated with cardiac hypertrophy.^40^ Assessment of these markers revealed significantly elevated levels of phosphorylated GSK-3β relative to total expression in the hearts of 21-week-old D2/*mdx* mice compared to all other groups (Figure 1I). However, there were no significant differences seen in AKT phosphorylation relative to total expression between groups (Figure 1J).

**Table 1:** Cardiac Cine-MRI Assessment of 14-, 21-, and 28-Week-Old BL10/WT, BL10/*mdx*, D2/WT, and D2/*mdx* Mice

			14 Weeks	21 Weeks	28 Weeks
**Left Ventricle**					

End diastolic volume	BL10	WT	68.51 ± 11.62	63.84 ± 5.54	60.31 ± 4.43
		*mdx*	66.25 ± 2.98	78.71 ± 5.77	77.41 ± 9.74
	D2	WT	49.24 ± 4.49	46.24 ± 5.94	50.25 ± 3.71
		*mdx*	47.44 ± 2.43	57.66 ± 1.96	54.03 ± 4.38
End systolic volume	BL10	WT	34.32 ± 10.74	22.95 ± 3.43	21.70 ± 2.99
		*mdx*	22.62 ± 2.03	26.85 ± 3.75	28.32 ± 5.92
	D2	WT	12.61 ± 2.11	18.13 ± 2.40	22.46 ± 3.86
		*mdx*	14.34 ± 2.71	14.89 ± 3.14	18.76 ± 2.83
Stroke volume	BL10	WT	34.28 ± 2.80	40.89 ± 4.01	38.61 ± 2.97
		*mdx*	43.63 ± 1.82*	51.86 ± 2.97	49.09 ± 6.01
	D2	WT	36.63 ± 3.22	28.11 ± 3.89	27.78 ± 3.62
		*mdx*	34.08 ± 1.68	42.77 ± 2.51*	35.28 ± 4.93
Ejection fraction	BL10	WT	53.76 ± 6.03	64.32 ± 4.22	64.34 ± 3.40
		*mdx*	66.06 ± 2.14	66.34 ± 2.44	65.65 ± 6.04
	D2	WT	74.77 ± 3.11	61.14 ± 2.53	55.36 ± 5.95
		*mdx*	64.18 ± 7.13	74.19 ± 4.95*	64.25 ± 6.04
Mass	BL10	WT	112.13 ± 17.98	116.58 ± 13.22	129.41 ± 5.81
		*mdx*	109.56 ± 8.00	124.49 ± 10.17	132.59 ± 14.39
	D2	WT	106.71 ± 5.82	100.27 ± 10.98	107.12 ± 12.02
		*mdx*	103.05 ± 12.64	131.60 ± 7.65*	135.05 ± 6.80

**Right Ventricle**					

End diastolic volume	BL10	WT	51.77 ± 4.39	50.31 ± 9.23	53.65 ± 4.89
		*mdx*	63.05 ± 2.26*	66.20 ± 7.32	74.17 ± 9.75
	D2	WT	63.41 ± 8.09	40.40 ± 8.43	39.75 ± 5.13
		*mdx*	52.13 ± 6.23	43.90 ± 6.82	40.12 ± 6.26
End systolic volume	BL10	WT	22.81 ± 5.66	18.28 ± 3.53	17.15 ± 1.66
		*mdx*	21.24 ± 1.84	23.64 ± 4.25	27.41 ± 3.62*
	D2	WT	14.98 ± 1.66	16.63 ± 3.97	18.76 ± 4.40
		*mdx*	20.19 ± 5.82	13.51 ± 3.27	16.62 ± 3.47
Stroke volume	BL10	WT	28.96 ± 1.40	32.03 ± 5.89	36.51 ± 4.97
		*mdx*	41.81 ± 1.24*	42.56 ± 5.41	46.76 ± 7.23
	D2	WT	48.43 ± 7.23	23.77 ± 4.88	20.99 ± 3.90
		*mdx*	31.94 ± 2.87	30.39 ± 4.65	23.49 ± 4.17
Ejection fraction	BL10	WT	57.97 ± 6.13	64.32 ± 2.64	66.97 ± 4.01
		*mdx*	66.55 ± 2.04	64.06 ± 4.80	62.40 ± 3.08
	D2	WT	75.50 ± 2.71	58.55 ± 3.23	52.89 ± 7.77
		*mdx*	63.49 ± 5.48	69.52 ± 3.65*	58.76 ± 5.42

All parameters are expressed as μL with the exception of ejection fraction (%) and mass (mg). Data represented as mean ± SEM, *p < 0.05 versus age-matched WT control, n = 6/group.

### Utrophin Is Elevated and Localized to the Sarcolemma in Striated Muscles of D2/*mdx* Mice administered AAV-μ-Utro

**Figure 2:**
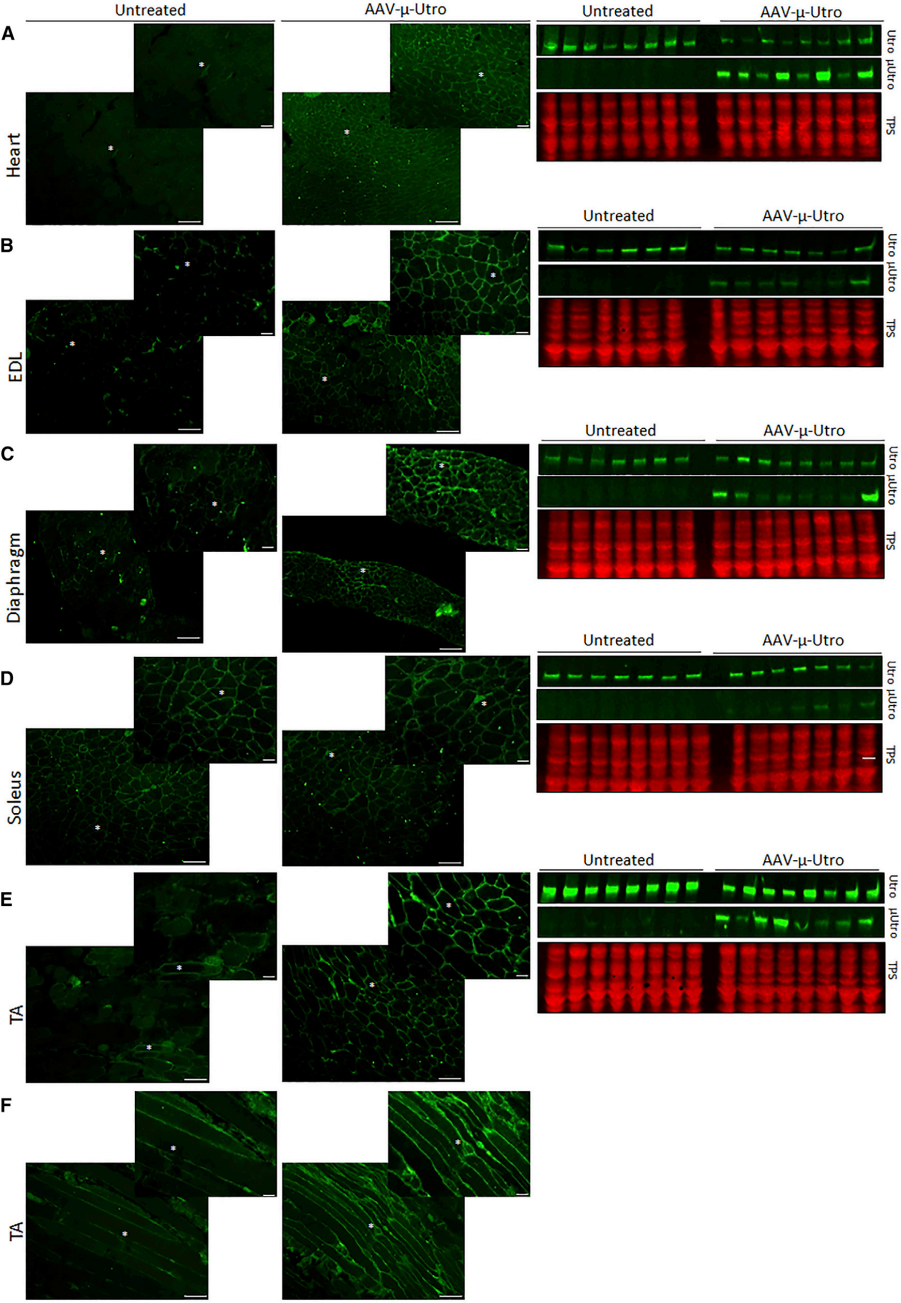
Utrophin Expression in 21-Week-Old D2/*mdx* Mice Administered AAV-μ-Utro Compared to Vehicle Control Mice Localization assessed by immunofluorescence and expression of full-length and micro-utrophin (Utro and μ-Utro, respectively) in cross-sections from (A) heart, (B) extensor digitorum longus (EDL), (C) diaphragm, (D) soleus, and (E) tibialis anterior (TA) muscles. Utrophin IF was also assessed in longitudinal section of TA muscles (F). Expression was assessed by western blot and shown relative to loading control (total protein stain; TPS). Scale bars, 100 μm, n = 8/group.

Western blot analysis revealed expression of the truncated utrophin protein in the cardiac muscle and in the fast type II tibialis anterior (TA) and extensor digitorum longus (EDL) skeletal muscles. Transgene expression was lower in the EDL muscles compared to the TA muscles (Figures 2B, 2E, and 2F). A lower level of truncated protein expression in diaphragm and the slow type I soleus muscle, which is more complicated to transduce, was noted. Quantification of μ-utrophin protein expression supports these observations (Figure S1A). The AAV genome copy number quantification (Figure S1B) confirmed the expected bio-distribution of the vector genome (vg), and results are in correlation with previous data generated with an AAV9.^41^ Immunofluorescence (IF) analysis revealed that utrophin was localized to the sarcolemma in cross-sections from the heart, diaphragm, EDL, and TA muscles from treated mice compared to vehicle control mice (Figures 2A–2E). Longitudinal sections from TA muscles also confirmed localization of utrophin to the sarcolemma along the myofibers (Figure 2F). Interestingly, weak or no correlation was evident between μ-utrophin protein expression or vector genome quantification and functional parameters in the EDL muscles, diaphragm muscle strips, or the heart (Figure S2).

### Contractile Properties and Architecture of Skeletal Muscles Were Improved in D2/*mdx* Mice Treated with AAV-μ-Utro Compared to Vehicle Control Mice

**Figure 3:**
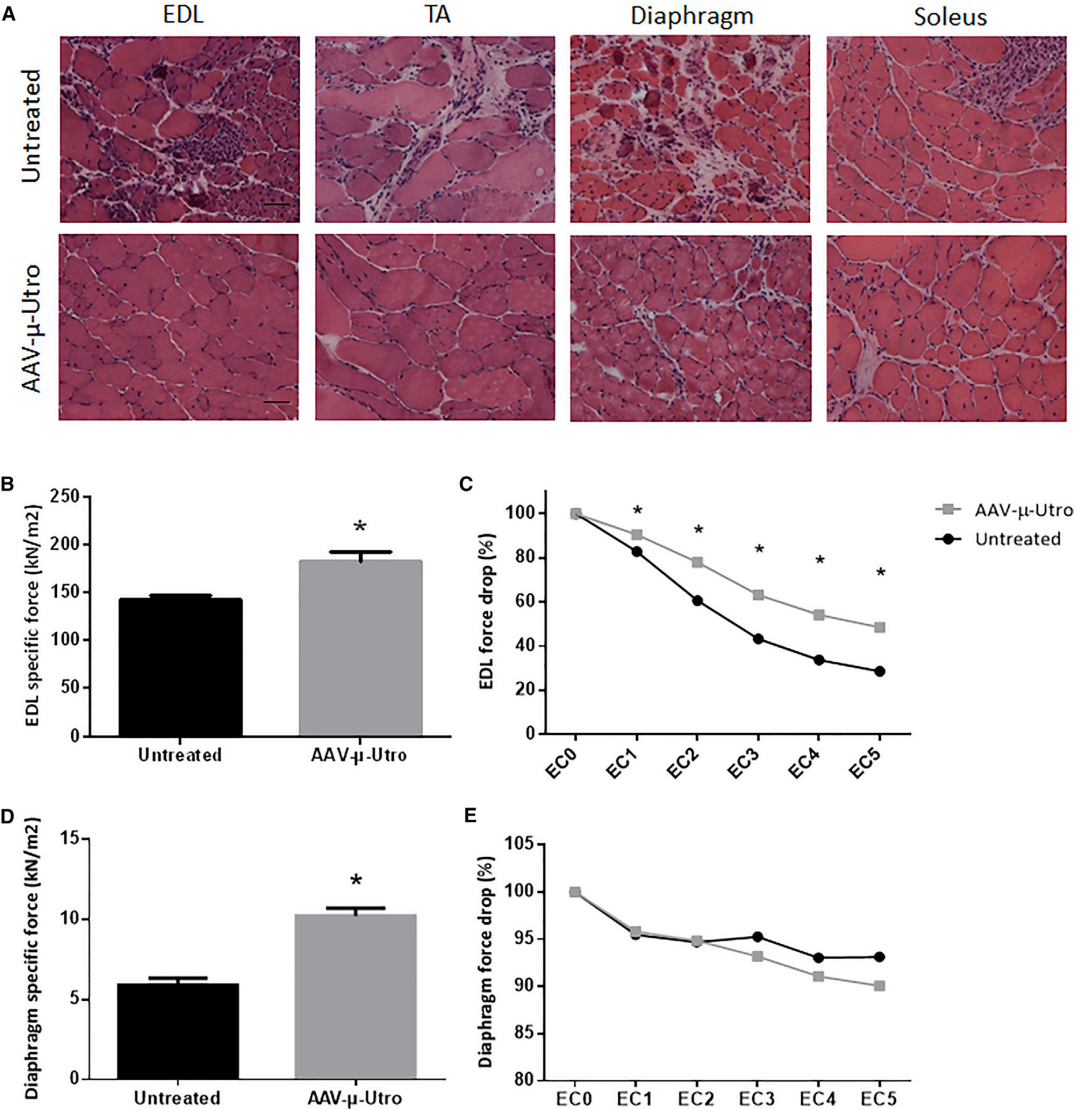
Skeletal Muscle Functional Testing and Muscle Architecture of D2/*mdx* Mice Administered AAV-μ-Utro Compared to Vehicle Control Mice (A) Muscle architecture assessed by H&E staining of cross-sections of EDL, tibialis anterior (TA), diaphragm, and soleus muscles from treated mice compared to vehicle control mice. (B) Specific force and (C) percentage force drop following 15% eccentric contraction in the extensor digitorum longus (EDL) muscles and (D) specific force and (E) percentage force drop following 15% eccentric contraction in diaphragm muscle strips from D2/*mdx* mice administered AAV-μ-Utro compared to vehicle control mice. Scale bar, 100 μm. Data represented as mean ± SEM, *p < 0.05 versus vehicle control mice, n = 8/group.

Muscle architecture was assessed by H&E and revealed improved muscle structure and reduced necrosis in EDL, TA, diaphragm, and soleus muscle cross-sections from D2/*mdx* mice administered AAV9/CMV-μ-utrophin (AAV-μ-Utro) compared to vehicle control mice (Figure 3A). *Ex vivo* muscle physiological testing of whole EDL muscles and diaphragm muscle strips was performed to determine the impact of treatment on skeletal muscle function. Despite low μ-utrophin protein expression observed, specific force of EDL muscles (Figure 3B) and diaphragm muscle strips (Figure 3D) was significantly greater in mice treated with AAV-μ-Utro compared to vehicle control mice. Percentage force drop was assessed following a series of eccentric contractions in the EDL muscles and diaphragm muscle strips from treated and vehicle control mice. Percentage force drop was significantly rescued in EDL muscles treated with AAV-μ-Utro and vehicle control mice following eccentric contraction (Figure 3C). Percentage force drop of diaphragm muscle strips following eccentric contraction was not different between groups (Figure 3E). Kyphosis score and muscle mass (TA, EDL, quadriceps, and soleus) were not altered with treatment (Table S2).

### Markers of Dystrophic Pathology Were Reduced in D2/*mdx* Mice Treated with AAV-μ-Utro Compared to Vehicle Control Mice

**Figure 4:**
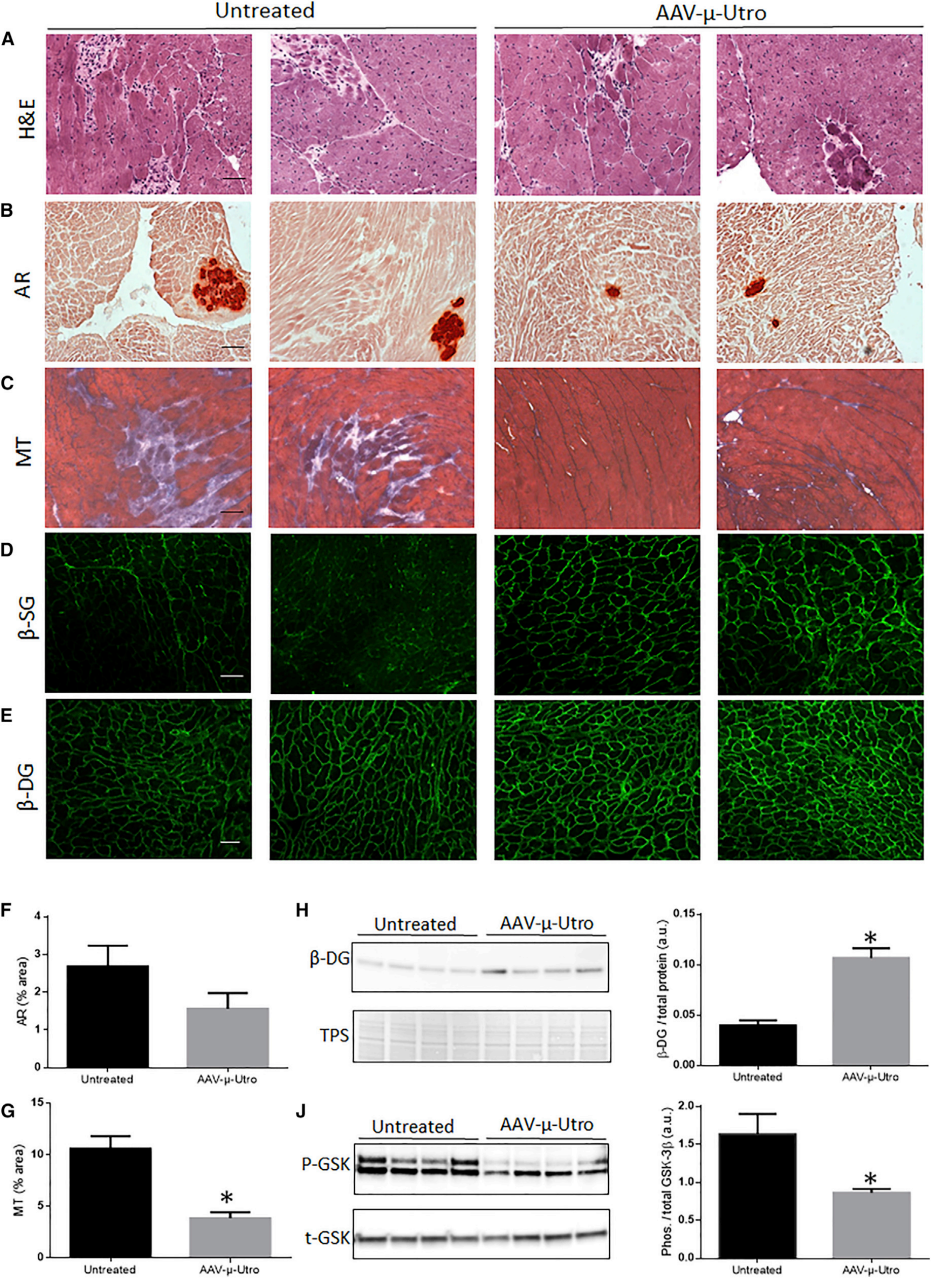
Assessment of Cardiac Pathology in D2/*mdx* Mice Administered AAV-μ-Utro Compared to Vehicle Control Mice Representative images of (A) muscle architecture (assessed by H&E), (B) calcification (red; assessed by alizarin red; AR), (C) fibrosis (assessed by Masson’s trichrome; MT; indicated in blue) of cardiac cross-sections from treated mice compared to vehicle control mice. Quantification of (F) AR and (G) MT, expressed as percentage area. Representative images and quantification of (H) β-dystroglycan (β-DG) and (J) phosphorylated and total GSK-3β (GSK) assessed by western blot. β-DG was normalized to loading control (total protein; TPS). Scale bar, 100 μm. Data represented as mean ± SEM, *p < 0.05 versus vehicle control mice, n = 8/group.

Cardiac cross-sections from treated and vehicle control mice were assessed for dystrophic pathology. Fewer dystrophic lesions were observed in the hearts of treated D2/*mdx* mice compared to vehicle control mice (Figure 4A). The hearts from D2/*mdx* mice treated with AAV-μ-Utro also showed lower levels of calcification and fibrosis compared to vehicle control mice (Figures 4B and 4C). However, only the reduction in fibrosis was statistically significant (Figures 4F and 4G). Components of the DGC are lost from the sarcolemma in dystrophic muscle and undergo proteolysis; therapies aimed at restoring dystrophin and modulating utrophin have shown to maintain these components. IF assessment of β-dystroglycan (β-DG) and β-sarcoglycan (β-SG) revealed a clear increase in localization of β-SG at the sarcolemma of AAV-μ-Utro-treated mice compared to vehicle control mice (Figure 4D). A slight increase of β-DG was also evident by IF assessment (Figure 4E), however to a lesser extent than β-SG. Western blot assessment was therefore also carried out and confirmed elevated levels of β-DG in the hearts of treated mice compared to vehicle control mice (Figure 4H). To assess the impact of treatment on hypertrophic signaling, phosphorylated GSK-3β was assessed by western blot. Reduced phosphorylation of GSK-3β was clearly evident in the hearts of D2/*mdx* mice administered AAV-μ-Utro compared to vehicle control mice (Figure 4J).

### AAV-μ-Utro Administration Ameliorates Hypertrophic Cardiomyopathy in D2/*mdx* Mice

**Figure 5:**
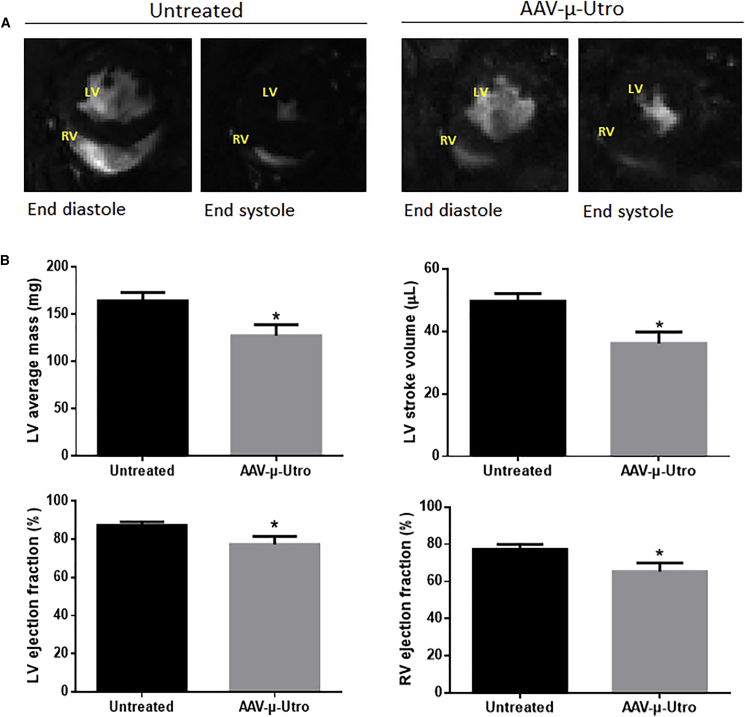
Cardiac Cine-MRI Assessment of D2/*mdx* Mice Administered AAV-μ-Utro Compared to Vehicle Control Mice (A) Representative cardiac cine-MRI short axis mid-papillary slices at end systole and diastole. Quantification of (B) left ventricle (LV) mass, LV stroke volume, LV ejection fraction, and right ventricle (RV) ejection fraction of D2/*mdx* mice administered AAV-μ-Utro compared to vehicle control mice. Data represented as mean ± SEM, *p < 0.05 versus vehicle control mice, n = 8/group. Left ventricle, LV; right ventricle, RV.

The elevated ejection fraction observed in the D2/*mdx* mice compared to age-matched D2/WT was reduced following AAV-μ-Utro treatment. Reduction in ejection fraction is evident in representative MRI short axis mid-papillary slices (Figure 5A). Quantitation of serial short axis slices revealed significant reductions in LV mass, LV ejection fraction, LV stroke volume, and RV ejection fraction in mice administered AAV-μ-Utro compared to vehicle control mice (Figure 5B). End diastolic volume, end systolic volumes (LV and RV), and RV stroke volume were not significantly attenuated with treatment (Table 2). Assessment of diastolic function revealed no difference between groups in peak ejection rate, peak filling rate, or time to peak ejection rate (Table 2).

**Table 2:** Cardiac Cine-MRI Analysis of 21-Week-Old D2/*mdx* Mice Administered AAV-μ-Utro Compared to Vehicle Control Mice

Systolic	Untreated	AAV-μ-Utro
**Left Ventricle**		

End diastolic volume (μL)	57.03 ± 3.27	46.70 ± 4.22
End systolic volume (μL)	7.43 ± 1.26	10.51 ± 1.76

**Right Ventricle**		

End diastolic volume (μL)	53.45 ± 9.73	52.60 ± 9.06
End systolic volume (μL)	12.14 ± 2.77	17.16 ± 3.51
Stroke volume (μL)	41.32 ± 7.66	35.44 ± 6.70

**Diastolic**		

Peak ejection rate	−605.50 ± 57.60	−412.76 ± 105.32
Peak filling rate (mL/s)	474.80 ± 60.21	448.72 ± 25.59
Time to peak ejection rate (mL/s)	8.50 ± 2.35	12.33 ± 4.24

Data represented as SEM, n = 8/group.

## Discussion

The data presented here show that AAV-μ-Utro administration ameliorates the skeletal and cardiac phenotype of D2/*mdx* mice, which present with a more severe pathology than the BL10/*mdx* mouse model due to differences in genetic background.^34^, ^35^ The positive effects we observed with μ-utrophin gene delivery in D2/*mdx* mice suggests that the modulation of utrophin will potentially be able to treat a large spectrum of disease presentations. Importantly, our data show that utrophin can replace dystrophin in the heart and improve cardiac function.

We demonstrated that 21-week-old D2/*mdx* mice exhibit a functionally significant hypertrophic cardiac phenotype compared to age-matched control mice. Due to the variation reported in the cardiac phenotype of D2/*mdx* mice,^35^, ^36^, ^41^ we conducted an extensive characterization of the model using cardiac cine-MRI. Cardiac cine-MRI was used, as it is far more accurate than other non-invasive techniques used for assessing cardiac function, such as echocardiogram.^38^ Our natural history studies in D2/*mdx* mice suggest that 21 weeks is a good time to study cardiac function. We found systolic parameters, ventricle mass, and heart mass to be significantly elevated in D2/*mdx* mice compared to D2/WT mice; these differences are indicative of hypertrophic cardiomyopathy. D2/*mdx* mice of this age have not before been assessed for cardiac function. Although myocardial calcification was observed in D2/WT mice, similar to previous studies,^41^ the cardiac histopathology was far more prominent in age-matched D2/*mdx* mice.

Interestingly, we observe a resolution in the cardiac phenotype by 28 weeks of age. Previous studies have also observed D2/*mdx* mice to present with a dynamic cardiac phenotype.^35^ DMD and more mildly affected Becker’s muscular dystrophy (BMD) patients have been shown to present with a hypertrophic phenotype that later progresses into the more classical dilated cardiomyopathy.^42^, ^43^ Given these observations in patients and that D2/*mdx* mice have been reported to present with a dilated cardiac phenotype, it is possible that the elevated systolic function we observed in 21-week-old D2/*mdx* mice could progress into a dilated phenotype later in life. Irrespectively, the prominent cardiac dysfunction and histopathology allow for the effective use of 21-week-old D2/*mdx* mice for assessing treatment efficacy.

We found that induction of μ-utrophin improved structure and function of skeletal muscles from D2/*mdx* mice. Following treatment, specific force output of diaphragm muscle strips and EDL muscles was greater from D2/*mdx* mice administered AAV-μ-Utro compared to vehicle control mice. Rescue of force drop following eccentric contraction was also evident in EDL muscles. In addition to functional benefits, assessment of skeletal muscle structure revealed improved architecture and reduced necrosis in the diaphragm and lower limb muscles of treated mice. Interestingly, there was no significant correlation between μ-utrophin levels and functional benefit. It may be that some transduced myofibers have broken down throughout the treatment period, partially contributing to improved function but not to final vector quantification. Further experiments are required to better understand AAV turnover in D2/*mdx* striated muscle. In agreement with previous studies, our results demonstrate that μ-utrophin elicits a functional benefit in dystrophin-deficient skeletal muscle.

Previously, modulation of truncated utrophin has shown to rescue skeletal muscle pathology of both BL10/*mdx* and *dko* mice.^31^, ^44^ Similarly, we observed in skeletal muscles of the D2/*mdx* model, that the μ-utrophin transgene is localized to the sarcolemma in contrast to the endogenous full-length utrophin that is found at the NMJ, NTJ, and regenerating myofibers. Therefore, the μ-utrophin transprotein can elicit a functional benefit even at low expression levels, irrespective of basal expression. Our findings show treatment efficacy in a model that is more severely affected than BL10/*mdx* mice and with a more relevant genomic context than *dko* mice. Efficacy of μ-utrophin treatment across all three pre-clinical contexts highlights its strong potential for treating an extensive range of patient pathology.

The hypertrophic cardiac phenotype evident in D2/*mdx* mice was rescued with AAV-μ-Utro-treatment. Systolic parameters, including LV and RV ejection fraction and LV stroke volume, were all attenuated toward WT levels. Although we observed a hypertrophic phenotype in D2/*mdx* mice, attenuation of systolic parameters with treatment validates this phenotype as pathological. The D2/*mdx* mouse model may therefore offer a unique pre-clinical context, as hypertrophic cardiomyopathy affects ∼26% of DMD patients^42^ and is currently not recapitulated pre-clinically. Reductions in fibrosis and calcification and preservation of the DGC were shown to underpin the functional benefits observed in hearts of AAV-μ-Utro-treated mice. Although we have previously shown that small compounds induce utrophin expression in cardiac muscle,^18^, ^19^ the data presented here are the first evidence of a functional cardiac benefit by direct utrophin upregulation. As D2/*mdx* mice present a unique cardiac context, further functional cardiac studies in other animal models such as the *dko* mouse model will be of interest.

Cardiomyopathy is a leading cause of death in DMD patients receiving assisted ventilation.^8^ Heart defects are evident in nearly all patients by the end of adolescence and are also present in more than 90% of BMD patients.^45^ Transgenic sarcospan upregulation and Tadalafil treatment have been shown to elicit utrophin expression and improve cardiac function.^46^, ^47^ However, the functional impact of direct utrophin upregulation on the dystrophic myocardium had not been investigated. The ability of μ-utrophin to improve cardiac function is therefore very exciting.

Interestingly, mini-dystrophin constructs that were shown to fully restore skeletal muscle function only partially rescued cardiac function in BL10/*mdx* mice.^23^ In a more recent study, a mini-dystrophin construct optimized for the heart more effectively rescued cardiac function in aged BL10/*mdx* mice.^48^ These studies highlight the importance of tissue-specific mini-gene construct design. It is also important to note that a fully optimized mini-gene will only produce a mild BMD phenotype in DMD patients. Approaches that can elicit expression of full-length utrophin, such as small drug treatment,^18^ in dystrophin-deficient hearts may have a clinically meaningful impact on the cardiomyopathy of DMD patients.

In summary, we have demonstrated the efficacy of AAV-μ-Utro for improving ambulatory, respiratory, and cardiac muscle function in severely affected D2/*mdx* mice. These data demonstrate that utrophin upregulation and localization to the sarcolemma of mature myofibers confers a functional benefit. Importantly, we show that μ-utrophin improves cardiac function of dystrophin-deficient hearts. These pre-clinical findings highlight the efficacy of μ-utrophin for both treating a broad spectrum of patient pathology and improving the critical respiratory and cardiac musculature. These data support the potential of full-length utrophin modulation as a powerful tool for treating skeletal muscle pathology and cardiomyopathy of all DMD patients whatever their underlying mutation.

## Materials and Methods

### Mice

All animal procedures were performed in accordance with UK Home Office regulations in compliance with the European Community Directive published in 1986 (86/609/ EEC). The study was performed under certificate of designation number 30/2306 and project license number 30/3104 following approval by the University of Oxford, Departments of Physiology, Anatomy & Genetics and Experimental Psychology Joint Departmental Ethics Review Committee. C57BL/10ScSnOlaHsd (BL10/WT), C57BL/10ScSn-*Dmd*^*mdx*^/J (BL10/*mdx*), D2/2J (D2/WT), and D2.B10-*Dmd*^*mdx*^/J (D2/*mdx*) male mice were assessed. D2/WT mice were obtained from Envigo (UK), and all other mouse strains were bred in the Biomedical Services facility, University of Oxford. 14-, 21- and 28-week-old mice were assessed for cardiac cine-MRI and then sacrificed. Muscles were immediately excised, and tendon and non-muscle material was removed and weighed on an analytical balance and snap frozen in liquid nitrogen or embedded in optimal cutting temperature (OCT) and frozen in thawing isopentane. Samples were stored at −80°C until further analysis.

### Treatment

AAV vector pseudotyped with serotype nine capsids was used for gene transfer of a murine codon-optimized μ-utrophin under the transcriptional control of the cytomegalovirus (CMV) promoter. The CMV promoter was used due to its comparable transduction of both skeletal and cardiac muscle.^21^ The μ-utrophin construct was a kind gift from Solid Biosciences and was modeled on optimized μ-dystrophin constructs.^28^ The complementary DNA encodes the N-terminal actin-binding domain, spectrin-like repeats 1–3, hinge region 2, the final spectrin-like repeat, and the dystroglycan-binding domain, as previously described.^31^ Four-week-old D2/*mdx* mice were administered 3 × 10^12^ vector genomes of AAV-μ-Utro by tail-vein injections, and vehicle control mice were administered the equivalent volume of PBS. Mice were then harvested at 21 weeks of age.

### Kyphosis Scoring

Kyphosis scoring is a quantitation of the severity of spinal curvature as determined by palpation of conscious mice based on a 1–5 index of kyphosis: 1 indicating no spinal deformity and 5 being the most severe.^49^, ^50^

### Cardiac Cine-MRI

As previously described,^51^, ^52^ cardiac cine-MRI was performed by anaesthetizing mice with 1.5% isoflurane in O_2_ followed by supine positioning in a purpose-built cradle. Electrocardiogram (ECG) electrodes were inserted into the forepaws and a respiration loop was taped across the chest. The cradle was lowered into a vertical-bore, 11.7 T MR System (Magnex Scientific, Oxon, UK) with a 40-mm birdcage coil (Rapid Biomedical, Würzburg, Germany) and a Bruker console running Paravision 2.1.1 (Bruker Medical, Ettlingen, Germany). A stack of consecutive 1-mm-thick short-axis ECG and respiration-gated cine-FLASH images (echo time/repetition time [TE/TR] 1.43/4.6 ms; 17.5° pulse; field of view 25.6 × 25.6 mm; matrix size 256 × 256; voxel size 100 × 100 × 1,000 μm; 20 to 30 frames per cardiac cycle) were acquired to cover the entire left and right ventricles of 14-, 21-, and 28-week-old mice. Image analysis was performed using ImageJ (NIH Image, Bethesda, MD). Ventricular mass and volumes were calculated as previously described.^53^ To allow for additional diastolic analysis, cardiac function of AAV-μ-Utro-treated mice and vehicle control mice were assessed on a 7 T Varian/Agilent pre-clinical MRI scanner. A 72-mm volume transmit coil (40-mm), 4-element surface receive array (both Rapid Biomedical, Würzburg, Germany) were used with a high temporal resolution following the approach as described previously.^49^ In brief, a single mid-papillary slice was acquired over two consecutive cardiac cycles, comprising 1-mm-thick short-axis cardiac gated cine-FLASH images (TE/TR 1.5/3.9 ms; 50-kHz bandwidth; 30° flip angle [Gaussian]; field of view 25.6 × 25.6 mm; matrix size 128 × 128, voxel size 200 × 200 × 1,000 μm; 30 to 50 frames per cardiac cycle) were acquired to cover the entire left and right ventricles. Multi-coil data was reconstructed in magnitude via sum of squares. Diastolic function was quantified as previously described.^49^

### Assessment of Skeletal Muscle Contractile Properties

Following cardiac cine-MRI analysis, mice were culled via CO_2_ overdose in accordance with schedule I of the UK Animals (Scientific Procedures) Act 1986 and contractile properties of the mouse EDL muscles were assessed *ex vivo*, as described previously.^54^ At the conclusion of these measurements, the TA, EDL, soleus, and quadriceps muscles were carefully excised, blotted on filter paper, and weighed on an analytical balance. The EDL muscle was mounted in embedding medium and frozen in thawing isopentane for later histochemical analyses. EDL muscle cross-sectional area was determined from the following equation: cross-sectional area = muscle mass/(L_f_ × 1.06), where L_f_ represents optimal fiber length and 1.06 represents the density of mammalian skeletal muscle.^55^ The entire diaphragm and rib cage were surgically excised, and costal diaphragm muscle strips composed of longitudinally arranged full-length muscle fibers were isolated and prepared for functional assessment *ex vivo*, as described previously.^20^ On completion of the functional analyses, the diaphragm muscle strip was trimmed of tendon and any non-muscle tissue, blotted once on filter paper, weighed on an analytical balance, mounted in embedding medium, and frozen in thawing isopentane for later histochemical analyses. Hearts were trimmed of atria, the mid-sections removed and mounted in embedding medium, frozen in liquid nitrogen-cooled isopentane, and stored at −80°C for histochemical analyses. The remaining two-thirds of the ventricles were frozen in liquid nitrogen and stored at −80°C for subsequent biochemical analyses.

### Histological Assessment

Serial cross-sections (10 μm) were cut through the hearts, diaphragm, EDL, soleus, and TA muscles using a refrigerated (−20°C) cryostat (Bright OTF5000 Cryostat, Casa Alvarez Scientific Material, Spain). To assess muscle architecture, fresh-frozen sections were reacted with H&E. Calcification was assessed by alizarin red staining, sections were incubated with 2% alizarin red S (Sigma-Aldrich [pH 4.2]) for 1–5 min and washed with distilled (d)H_2_O and cleared. To assess fibrosis in the heart, cardiac cross-sections were stained with Masson’s trichrome as described previously.^49^ All sections were air-dried before application of coverslip and mounting medium and acquisition of digital images (Axioplan 2 Microscope System; Carl Zeiss, Germany). Quantified of alizarin red and Masson’s trichrome was performed using Fiji ImageJ 1.49i software and expressed as percentage area.

### Western Blot Analysis

Muscle samples were homogenized (Polytron 2100; Lucerne, Switzerland) for 3–15 s on ice in radioimmunoprecipitation assay (RIPA) buffer (Sigma-Aldrich) supplemented with protease inhibitors (1:100; Sigma-Aldrich) and sodium orthovanadate (1 mM; Sigma-Aldrich). Following normalization of protein concentration, homogenates were resolved in Laemmli buffer before heating to 95°C for 5 min and separated by SDS-PAGE. For utrophin detection, samples were loaded onto NuPAGE 3%–8% TRIS Acetate Midi Gels (Novex, Life Technologies) and transferred overnight to polyvinylidene fluoride (PVDF) membranes (Millipore). Membranes were blocked for 1 hr in Odyssey Blocking buffer (LI-COR; USA; 926-41090) and then incubated with primary antibodies overnight at 4°C. Primary antibody and dilutions used were as follows: utrophin, (MANCHO3; 84A) 1:50, a gift from G.E. Morris, and utrophin A (developed in-house as previously described) 1:200.^14^ Membranes were incubated with secondary antibodies diluted in Odyssey Blocking buffer for 1 hr at room temperature. Secondary antibody dilutions were as follows: IRDye 800CW donkey anti-mouse (LI-COR Biosciences; 926-32212) 1:5,000 and IRDye 800CW donkey anti-rabbit (LI-COR Biosciences; 926-32213) for utrophin and utrophin A, respectively. Infrared fluorescence of the secondary antibodies was read on an Odyssey Imaging System (LI-COR Biosciences; USA). The REVERT Total Protein Stain (LI-COR Biosciences; 926-11011) was used to control equal total protein loading. For all other targets, samples were loaded onto Criterion TGX (Tris-Glycine eXtended) pre-cast gels (Bio-Rad Laboratories, CA, USA) and transferred for 2 hr to PVDF membranes (Millipore). Membranes were blocked for 1 hr with 5% BSA (Sigma) in 0.1% Tris-buffered saline Tween (TBS-T) and then incubated with primary antibodies in 5% BSA in 0.1% TBS-T overnight at 4°C. Primary antibodies and dilutions used were as follows: phosphorylated GSK-3β (Cell Signaling; 9331S) 1:1,000, GSK-3β (Cell Signaling Technologies; 9315S) 1:1,000, phosphorylated (Ser473) AKT (Cell Signaling Technologies; 9271S) 1:1,000 and AKT (Cell Signaling; 9272S) 1:1,000, and β-dystroglycan (DG; Leica Biosystems; NCL-b-DG). Antibody binding was detected with horseradish peroxidase (HRP)-conjugated immunoglobulin and visualized by chemiluminescent detection (ECL Prime Western Blotting detection reagent Amersham) and imaging system ImageQuant LAS 4000 (GE Healthcare Life Sciences). Band densities were quantified with the Fiji ImageJ 1.49i software and normalized to total protein content of samples. Total protein was assessed by incubating membranes in Ponceau S solution (Sigma-Aldrich) followed by imaging using the ImageQuant LAS 4000 (GE Healthcare Life Sciences). Total protein staining was used as the loading control as housekeeping genes are not stable across genotypes.

### qPCR Analysis

Total RNA was extracted from 10 to 20 mg of TA muscle using TRIzol reagent as per manufacturer’s recommendations. 250 ng RNA was transcribed into cDNA using the QuantiTect Reverse Transcription kit (QIAGEN: 205313), and the resulting cDNA was stored at −20°C for subsequent analysis. qPCR was performed using the StepOnePlus Real-Time PCR system (Applied Biosystems) with SYBR Fast Master Mix (Thermo Fisher; 4385612). Results were analyzed using the ΔΔCT method. Quantification of the AAV genome copy number was performed using probes targeting the CMV promoter. The copy number standard curve of known amount of the AAV-μ-Utro plasmid was used to convert the threshold cycle (Ct) value of each reaction to the vg copy number. The data are reported as the vg copy numbers per diploid genome in each tissue.

### Immunofluorescence

For assessment of utrophin, fresh frozen cardiac cross-sections (10 μm thick) were blocked for 30 min with 10% fetal bovine serum (FBS)/PBS and then incubated with primary antibodies in 5% FBS and PBS for 2 hr at room temperature. For assessment of β-DG and β-SG, a mouse on mouse (MOM) detection kit (BMK-2202; Vector Laboratories; USA) was used per manufacturer’s instructions. Primary antibodies and dilutions used were as follows: utrophin A (developed in-house as previously described^14^) 1:2,000, β-SG (Novocastra; NCL-L-b-SARC) 1:50, and β-DG (Novocastra; NCL-b-DG) 1:100. Sections assessed for utrophin were reacted with Alexa Fluor 488 donkey anti-goat IgG antibody (Thermo Fisher Scientific; A-11055) 1:2,000 for 1 hr at room temperature. Sections assessed for β-SG and β-DG were reacted with Alexa Fluor 488 goat anti-mouse IgG antibody (Life Technologies; ab150117) 1:100 for 1 hr at room temperature. All sections were rinsed in PBS before air drying and application of coverslip with fluorescent mounting medium. Intensity was detected using a fluorescence microscope (Axioplan 2 Microscope System; Carl Zeiss, Germany). Utrophin staining, imaging, and image selection were performed blind.

### IgG Staining for Assessment of Membrane Integrity

Fresh-frozen muscle cross-sections were blocked for 30 min with 10% FBS and PBS and then incubated with Alexa Fluor 488 goat anti-mouse IgG (Life Technologies; ab150117) antibody, 1:750 in 5% FBS and PBS overnight at 4°C. Sections were rinsed in PBS before air drying and application of cover with fluorescent mounting medium; intensity was detected using a fluorescence microscope (Axioplan 2 Microscope System; Carl Zeiss, Germany).

### Statistical Analysis

Results were analyzed using Prism (GraphPad Software). Statistical comparisons were made between age-matched and parent-strain matched (i.e., BL10/WT versus BL10/*mdx* and D2/WT versus D2/*mdx*) groups and between AAV-μ-Utro-treated and vehicle control mice. A non-parametric t test was used to determine significant differences between groups, with the level of significance set at p < 0.05 for all comparisons. Linear regression analysis was performed to determine correlations between groups. All values are presented as mean ± SEM.

## Author Contributions

S.G., T.L.K., and K.E.D. conceived and designed experiments; T.L.K., S.G., B.E, S.S., L.M., and A.B. performed the experiments; T.L.K., S.G., B.E., and K.E.D. analyzed the data; T.L.K., S.G., and K.E.D. wrote the paper; G.O. and J.S.C. characterized the mini-utrophin gene; D.G. and J.S. contributed support and drafting advice on the manuscript. All authors edited the paper and approved the submission.

## Conflicts of Interest

K.E.D. is a co-founder and member of the Scientific Advisory Board of Summit Therapeutics. D.G. and J.S. are employees of Solid Biosciences. J.S.C. is on the Scientific Advisory Board of Solid Biosciences.

## Supplemental Information

Document S1. Figures S1 and S2 and Tables S1 and S2

Document S2. Article plus Supplemental Information
