# Supplementary material for: Micro-utrophin Improves Cardiac and Skeletal Muscle Function of Severely Affected D2/mdx Mice
Source: Mol Ther Methods Clin Dev. 2018 Oct 16;11:92–105. doi: 10.1016/j.omtm.2018.10.005 (PMC6216100; doi:10.1016/j.omtm.2018.10.005)
Supplement: Document S1. Figures S1 and S2 and Tables S1 and S2 [file mmc1.pdf]

OMTM, Volume *11*

Supplemental Information

Micro-utrophin Improves Cardiac and Skeletal Muscle Function of Severely Affected D2/*mdx* Mice

Tahnee L. Kennedy, Simon Guiraud, Ben Edwards, Sarah Squire, Lee Moir, Arran Babbs, Guy Odom, Diane Golebiowski, Joel Schneider, Jeffrey S. Chamberlain, and Kay E. Davies

Supplemental Figures

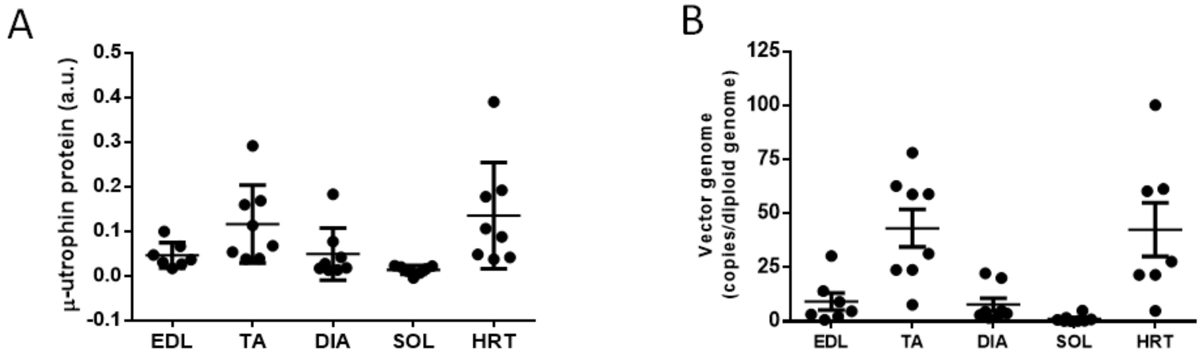

Supp. Figure 1.

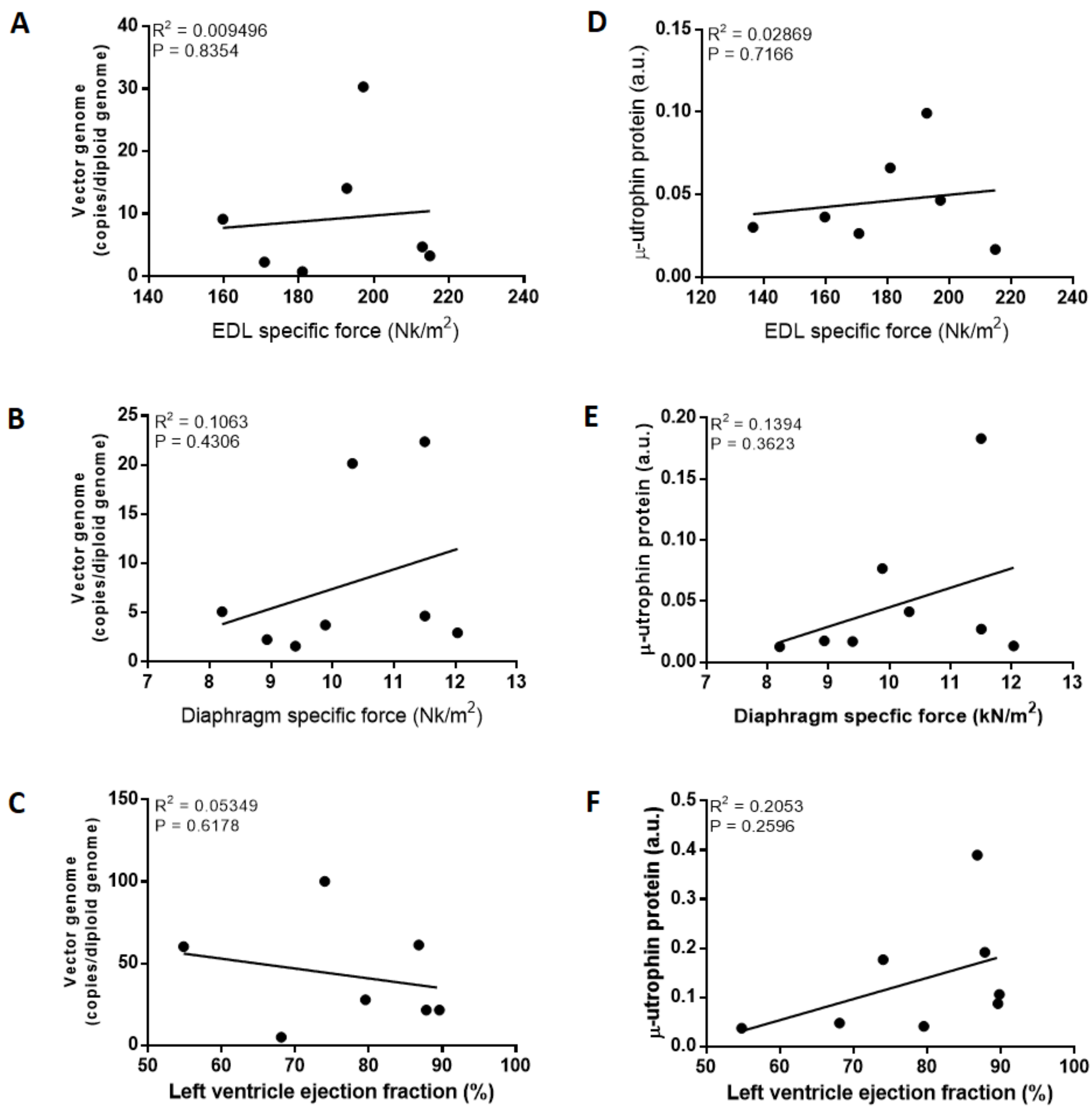

Supp. Figure 2.

Supplemental Figure Legends

Supp. Figure 1. Quantification of µ-utrophin protein expression and vector genome copy number in cardiac and skeletal muscles of D2/*mdx* mice administered AAV-µ-Utro. Quantification of (**A**) µ-utrophin protein expression and (**B**) qPCR detection of the CMV promoter in cardiac and skeletal muscles from D2/*mdx* mice administered AAV-µ-Utro. Data represented as mean ± SEM, n = 8/group. EDL, extensor digitorum longus; TA, tibialis anterior; DIA, diaphragm; SOL, soleus; HRT, heart.

Supp. Figure 2. Assessment of the linear relationship between µ-utrophin protein expression or vector genome expression and functional parameters of cardiac and skeletal muscles from D2/*mdx* mice administered AAV-µ-Utro. Assessment of the liner relationship between µ-utrophin protein expression and (**A**) specific force extensor digitorum longus (EDL) muscles, (**B**) specific force of diaphragm muscle strips and (**C**) left ventricle ejection fraction of D2/*mdx* mice administered AAV-µ-Utro. Assessment of the linear relationship between vector genome expression and (**D**) specific force of EDL muscles, (**E**) specific force of diaphragm muscle strips and (**F**) left ventricle ejection fraction of D2/*mdx* mice administered AAV-µ-Utro. No statistically significant correlations was observed between parameters. n = 8/group.

Supplementary Table 1. Body mass, kyphosis score and muscle mass of 14-, 21- and 28-week BL10/WT, BL10/*mdx*, D2/WT and D2/*mdx* mice

			14 weeks	21 weeks	28 weeks
**Body mass (g)**	**BL10**	**WT**	**28 ± 1**	**30 ± 1**	**33 ± 2**
		mdx	***35 ± 1 ****	**34 ± 2**	***39 ± 1 ****
	D2	WT	28 ± 1	33 ± 1	31 ± 2
		mdx	24 ± 1	*27 ± 1 **	30 ± 2
**Kyphosis score**	**BL10**	**WT**	**1 ± 0**	**1.1 ± 0.1**	**1 ± 0**
		mdx	**1 ± 0**	**1.3 ± 0.2**	*2.1 ± 0.2 **
	D2	WT	1.7 ± 0.1	2.8 ± 0.2	3.4 ± 0.1
		mdx	2.2 ± 0.2 *	2.2 ± 0.1	3.2 ± 0.2
**HEART**					
**Heart mass (mg)**	**BL10**	**WT**	**189.6 ± 23.3**	**138.1 ±2.8**	**146.8 ± 7.2**
		mdx	***227.1* ± 7.2**	***152.3 ± 3.2 ****	**155.1 ± 3.0**
	D2	WT	155.3 ± 18.6	155.9 ± 3.4	153.3 ± 4.9
		mdx	182.1 ± 13.5	*153.0 ± 4.7*	162.7 ± 8.1
**Heart/body mass (mg/g)**	**BL10**	**WT**	**6.7 ± 0.7**	**4.7 ± 0.2**	**4.4 ± 0.2**
		mdx	**6.5 ± 0.3**	**4.3 ± 0.2**	**4.0 ± 0.1**
	D2	WT	5.8 ± 0.9	4.6 ± 0.1	5.1 ± 0.3
		mdx	7.6 ± 0.2	*5.8 ± 0.3 **	5.5 ± 0.2
**SKELETAL MUSCLE**					
**TA/body mass (mg/g)**	**BL10**	**WT**	**1.57 ± 0.38**	**1.45 ± 0.05**	**1.30 ± 0.60**
		mdx	***2.20 ± 0.08 ****	***2.29 ± 0.10 ****	***2.17 ± 0.06 ****
	D2	WT	1.39 ± 0.90	1.13 ± 0.05	1.19 ± 0.07
		mdx	1.52 ± 0.14	1.31 ± 0.09	1.19 ± 0.08
**EDL/body mass (mg/g)**	**BL10**	**WT**	**0.27 ± 0.026**	**0.32 ± .01**	**0.30 ± 0.01**
		mdx	***0.45 ± 0.02 ****	***0.47 ± 0.02 ****	***0.45 ± 0.01 ****
	D2	WT	0.33 ± 0.06	0.26 ± 0.01	0.29 ± 0.01
		mdx	0.23 ± 0.26	0.31 ± 0.06	0.26 ± 0.03
**Quad/body mass (mg/g)**	**BL10**	**WT**	**5.18 ± 0.73**	**6.19 ± 0.15**	**5.43 ± 0.51**
		mdx	***9.05 ± 0.31 ****	***9.77 ± 0.27 ****	***8.97 ± 0.21 ****
	D2	WT	5.12 ± 0.34	4.99 ± 0.32	5.58 ± 0.24
		mdx	4.29 ± 0.26	*3.96 ± 0.14 **	*3.54 ± 0.22 **

TA, tibialis anterior; EDL, extensor digitorum longus; Quad, quadriceps. Data represented as mean ± SEM, *$P < 0.05$ vs age-matched WT control, $n = 6$/group.

Supplementary Table 2. Body mass, kyphosis score and muscle mass of 21-week-old D2/*mdx* mice administered AAV-μ-Utro compared to vehicle control mice.

	**Untreated**	**AAV-μ-Utro**
**Body mass (g)**	**28 ± 1**	**26 ± 1**
Kyphosis score	2.5 ± 0.2	2.3 ± 0.2
**HEART**		
**Heart mass (mg)**	**165.9 ± 7.7**	**156.0 ± 8.3**
Heart mass/body mass (mg/g)	6.0 ± 0.3	6.0 ± 0.2
**SKELETAL MUSCLE**		
**TA/body mass (mg/g)**	**2.0 ± 0.1**	**1.8 ± 0.8**
EDL/body mass (mg/g)	0.3 ± 0.0	0.3 ± 0.0
**Quad/body mass (mg/g)**	**4.6 ± 0.3**	**6.2 ± 0.3**
Sol/body mass (mg/g)	0.6 ± 0.1	0.3 ± 0.0

Data represented as mean ± SEM, $n = 8$/group. No statistical differences were observed between groups. Tibialis anterior, TA; extensor digitorum longus, EDL; quadriceps, Quad; soleus, Sol.
